# Array Comparative Genomic Hybridizations: Assessing the ability to recapture evolutionary relationships using an *in silico *approach

**DOI:** 10.1186/1471-2164-12-456

**Published:** 2011-09-21

**Authors:** Luz B Gilbert, Lee Chae, Takao Kasuga, John W Taylor

**Affiliations:** 1 Laboratoire de Recherche en Sciences Végétales, UMR CNRS-Université Paul Sabatier 5546, Chemin de Borde Rouge - Auzeville 31326, Castanet Tolosan, France; 2 Department of Plant Biology, Carnegie Institution for Science, Stanford, California 94305, USA; 3 USDA ARS, Plant Pathology Department, UC Davis, Davis, CA, 95616, USA; 4 Department of Plant and Microbial Biology, University of California Berkeley, Berkeley CA 94720, USA

## Abstract

**Background:**

Comparative Genomic Hybridization (CGH) with DNA microarrays has many biological applications including surveys of copy number changes in tumorogenesis, species detection and identification, and functional genomics studies among related organisms. Array CGH has also been used to infer phylogenetic relatedness among species or strains. Although the use of the entire genome can be seen as a considerable advantage for use in phylogenetic analysis, few such studies have questioned the reliability of array CGH to correctly determine evolutionary relationships. A potential flaw in this application lies in the fact that all comparisons are made to a single reference species. This situation differs from traditional DNA sequence, distance-based phylogenetic analyses where all possible pairwise comparisons are made for the isolates in question. By simulating array data based on the *Neurospora crassa *genome, we address this potential flaw and other questions regarding array CGH phylogeny.

**Results:**

Our simulation data indicates that having a single reference can, in some cases, be a serious limitation when using this technique. Additionally, the tree building process with a single reference is sensitive to many factors including tree topology, choice of tree reconstruction method, and the distance metric used.

**Conclusions:**

Without prior knowledge of the topology and placement of the reference taxon in the topology, the outcome is likely to be wrong and the error undetected. Given these limitations, using CGH to reveal phylogeny based on sequence divergence does not offer a robust alternative to traditional phylogenetic analysis.

## Background

The field of comparative genomics, particularly in microbes, has benefited greatly by the proliferation of whole genome sequences. The advantages of having sequences from multiple, related organisms range from improving annotation to characterizing the genetic basis of major phenotypic differences between strains or species [1-4]. While there are organisms for which multiple sequences from several different strains or species are available http://www.genomesonline.org/, in many cases resource limitations restrict the number of sequencing projects for members of the same genus. An appealing alternative to characterize sequence polymorphisms among related organisms is array Comparative Genome Hybridization (array CGH). This technique is attractive because microarrays made from genome sequence or even random DNA fragments from just one individual can be used to study the phylogenetic relationships among many closely related species.

Array CGH (aCGH) for two color array platforms uses DNA samples from a reference individual and a test individual, each labelled with a different fluorescent dye, and competitively hybridizes them to an array composed of immobilized DNA fragments from the reference individual [5-8]. This technique has primarily been used to characterize gene copy number changes and deletion events and has been applied extensively in the study of human tumorogenesis and bacterial pathogens [9-14].

The relative ease with which aCGH provides large amounts of discriminating information between individuals makes it a very attractive technique to determine relatedness. Comparisons between aCGH derived trees and trees based on DNA sequence of one or a few loci have supported this assumption [15-18]. However, the commonly used ribosomal DNA sequences do not necessarily provide enough resolution at the species and subspecies level to accurately resolve a tree [19,20]. Indeed, it has been shown that a detailed multi-locus phylogenetic analysis is often necessary to accurately resolve a species tree [21-27].

Practitioners of aCGH have inferred evolutionary relationships using distance methods implemented by programs such as Cluster, Genespring, and Acuity [28-30]. Several studies have used aCGH to compare bacterial pathogens and infer relatedness of strains from traditional cluster analysis [31-37]. A recent study utilized both human and bovine clustered aCGH data to make inferences on evolutionary relatedness [38]. Other studies have applied distance and parsimony techniques to build phylogenies from their array data [17,39-44]. Another compared MLST to aCGH trees derived by Bayesian inference [37].

**Figure 1:**
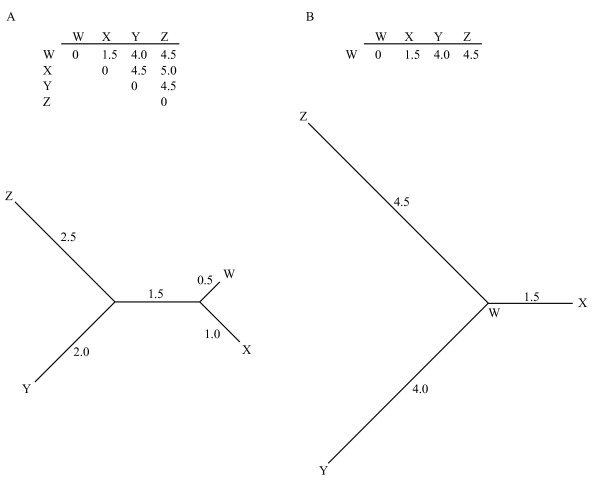
**The single reference design**. 1A shows an example of a pairwise distance matrix for the tree below the matrix. In 1B the distances of other taxa to W, typical of a single reference design. Below it is the star topology constructed from these distances when no other information is available to differentiate distances among X, Y, and Z.

A complicating factor not addressed in these studies is that typically a single individual is represented on an array. Having a single reference species or strain is at odds with traditional sequence based phylogenetic distance methods, where all pairwise comparisons among the taxa are used in constructing a species tree. This difference is illustrated in Figure 1. Having a single reference (Figure 1B) provides direct comparisons between all taxa and the reference taxon W, but provides no direct information about the relationships among the non-reference taxa X, Y, and Z. The assumption made for array CGH phylogeny is that the massively parallel nature of the technique provides sufficient evolutionary signal to infer the true relationships between all the taxa, in a manner similar to clustering analysis of gene expression data [28]. A further complication is that genes absent in the reference strain cannot be included in the analyses.

In this study we ask, what effect does having a single reference taxon have on recovering phylogenetic relationships? What effects do varying amounts of sequence divergence among taxa have on recovering the "true" tree? What impact do different tree topologies have? Does the position of the reference taxon in that topology have any importance or do all taxa serve equally well as the reference? Are distance and parsimony tree construction methods equivalent when applied to this type of data?

In order to address these questions, we chose to simulate CGH array data to avoid the added complexity of experimental noise associated with microarray technology. We chose the *Neurospora crassa *genome as a starting point for our simulated data, and evolved the sequence based on one of three simple topologies using empirically determined rates of nucleotide substitution. We modelled a very conservative system- allowing only nucleotide substitution, not gene gain or loss as that would complicate the tree-building process to generate optimal data with a high but realistic amount of phylogenetic signal. This *in silico *approach allowed us to vary all of the parameters necessary to test these questions, e.g., tree topology, position of reference taxon, and mean substitution rate and its variation, which would be impossible with any single set of empirical data.

## Methods

### CGH Data Simulation

We chose 70 mer sequences to represent the length of probes in a standard long oligo array platform because they have been a popular choice for groups investing in arrays for their chosen organism [7,45,46]. From the genome sequence of the filamentous fungus *Neurospora crassa*, 70 mer oligonucleotides were designed to provide hybridization probes for each of the 10,200 ORFs in the release 3 annotation, with an additional 300 probes devoted to non-coding regions [47-50]. This set of probe sequences was designated as the ancestral sequence and, along with a phylogenetic tree specifying the relationships and genetic distances among the taxa, was input into the sequence evolution program ROSE v1.3 to evolve sequences for related taxa [51].

Because not all genes in a genome evolve at the same rate, empirical data were analyzed to determine an appropriate distribution of sequence divergence to evolve our sequences. Based on the whole genome comparisons for different yeast species [4,52], a normal curve with a range of average polymorphisms was approximated to model sequence heterogeneity among related taxa across all coding regions. Three values of polymorphism appropriate for the detection limits of a 70 mer array, were chosen to model the distribution, 5 ± 1.44%, 7.5 ± 2.2%, and 10 ± 2.9%. The standard deviation of this distribution was empirically derived from the publicly available comparative genome analysis of different yeast species [1].

To simulate variation in evolutionary rate among genes, for each 70 mer, the branch lengths of the input tree were multiplied by a scaling factor, randomly drawn from the distribution of evolutionary rates. By varying the mean and standard deviation of the distribution of evolutionary rates, we varied the amount of evolutionary distance between the taxa of our chosen tree topology. These parameters were used to evolve sequences for each of 10,500 genes for each taxon using the Jukes-Cantor model of evolutionary change, allowing nucleotide substitution but not indels. This simple model of evolutionary change models a scenario where species are close relatives and the problem of multiple substitutions at single nucleotide positions is minimal, i.e. where the signal to noise ratio is highest. The genetic distances between all pairs of taxa were calculated for each locus using dnadist from the PHYLIP package v3.6 [53]. These distances are used to approximate DNA-DNA hybridization levels.

**Figure 2:**
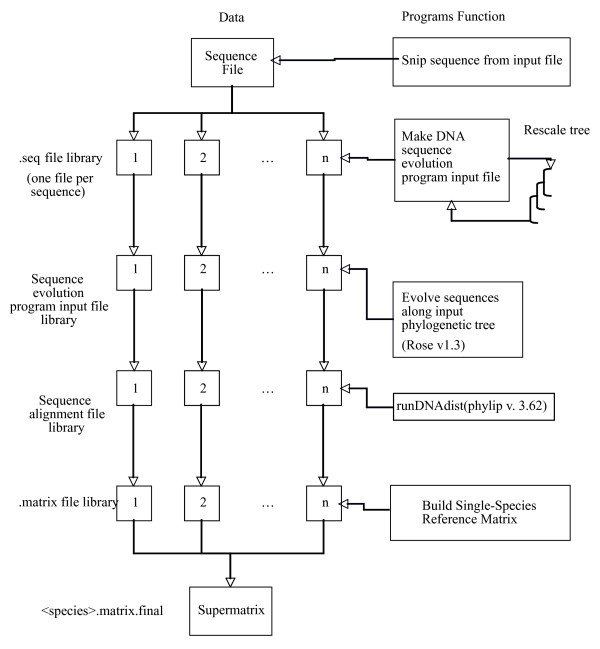
**Program Flow**. The diagram indicates the simulation program flow used to construct the supermatrix, our simulated CGH data matrix. Variables that can be modified include the number of sequences input, the topology of the tree used, and the amount of divergence among taxa in the simulated alignment, and the choice of evolutionary model to evolve and calculate genetic distance among taxa (see methods). Neighbor-Joining (NJ) and Parsimony Majority-Rule (PMR) trees were constructed from the supermatrix.

To simulate the use of a microarray based on a single reference taxon, one species from each tree was chosen as the reference and only those pairwise comparisons involving the reference individual were saved, corresponding to the genetic distance from the reference species to all other taxa. These distances were combined into one supermatrix with 10,500 genes for *n *taxa. This process is illustrated in Figure 2. This supermatrix represents the experimental design of a typical single-reference CGH experiment and was the basis for phylogenetic tree construction using PAUP version 4.0b10 for unix [54]. Additionally, as a control, sequence alignments for all taxa were concatenated and used to calculate, using PAUP, both distance based and parsimony trees.

To evaluate the effect of using much longer oligomers to construct an array, as with cDNA arrays, a limited number of simulations were conducted for probes of 500 bases. The results using the longer arrays were not substantially different from those of the 70 base trials (see supplemental data).

### Tree Construction

To construct a distance tree, a Pearson correlation-based distance matrix and a Euclidean distance matrix were derived from the original supermatrix using the statistical package R (http://www.R-project.org, R Development Core Team, 2006). Neighbor-joining was used to make phylogenetic trees based on these matrices, employing the minimum evolution criteria in PAUP [54]. To convert simulated DNA hybridization levels to characters with discrete states (0,1), simulated hybridization levels above the third quartile, representing the most divergent genes, were converted to 0 and those below to 1. This simple discretization method was amongst the most powerful when considering various gene categorization methods in our accompanying experimental analysis and was the simplest to implement *in silico*. Maximum parsimony was then used to infer phylogenetic tree topologies [54].

### Tree to tree distance metric quantification

To assess the outcome of a simulation, we compared the phylogenetic tree used to produce the data sets to the phylogenetic tree recovered from the CGH simulation. Initially, we measured the differences between the input tree and that produced by the simulation with two tree-to-tree distance metrics - symmetric distance and agreement subtree - implemented in PAUP [54]. Symmetric distance (SymD) determines the number of branches that must be rearranged or collapsed to make two topologies identical. If the topologies are identical and no branches need to be moved, the step size is 0. One step indicates a single collapsed branch and a rearrangement between two taxa is scored as two steps [55]. The agreement subtree metric, D1, counts the number of taxa that must be pruned from the trees make their topologies identical [56].

### Cophenetic correlation distributions

Neither the symmetric nor the agreement subtree metric compares branch lengths between the input and output trees. To investigate differences in branch length, we calculated the cophenetic correlation coefficient (CCC, from the cophenetic.phylo and cor functions implemented in the R stats and ape packages [57,58]), which measures how well a tree fits the data used to create it on a scale of 0 to 1, with 1 indicating a complete fit.

In this work, we used the CCC to compare the tree topology used to initiate the simulated data set (input tree) with the distance matrices resulting from the various CGH analyses. The R functions extrapolate distances from the branch lengths from the starting topology to compare to the distance matrix estimated by the simulation. The converse comparisons, where tree topologies resulting from simulations are compared to a distance matrix calculated from the starting topology, were very similar to the first comparisons and are not presented here.

## Results

The efficacy of CGH as a phylogenetic method as judged by two metrics (SymD or D1) varied considerably with topology (balanced, pectinate or empirical *Neurospora*), tree building algorithm (NJ with Pearson or Euclidian distances, or parsimony), and location of the reference taxon relative to the other taxa (basal or derived positions). The numerical scores for both tree-building methods are provided as Additional Files 1 and 2: Tables S1 and S2.

The parameter with the least effect was substitution rate (5%, 7.5% or 10%). The simplest topology, the balanced tree, was recovered most successfully. This special case is not likely to be found in nature, and it should be noted that permuting the branches individually may lead to different results. For the other two topologies, the mixture of close and more distantly related taxa presented a complication for the different tree-building algorithms. For both the pectinate topology and the *Neurospora *phylogeny, the relationship of the reference taxa to its sister taxon was not consistently well resolved. For the pectinate tree, the output tree differed from the input tree from between 0 to 8 steps (SymD) or 0 to 2 for distance D1, with scores growing progressively worse based on the choice of reference taxon. For the *Neurospora *topology, results also vary for the different reference positions and for the various tree-building algorithms, with step differences between input and output trees of 0 to 10, (SymD) or 0 to 4 (D1).

Generally, the parsimony trees were more sensitive to the changes in average nucleotide substitutions than those made by the Neighbor-Joining method. When sequence divergence was high (10%), parsimony phylogenies were poorly resolved. At 10% divergence, we checked the effect of changing the threshold for considering a hybridization difference to be diverged, that is, from 1 (present) to 0 (absent). Altering the threshold from 75% (where the most diverged 25% of the genes were given a score of 0) to 20%, 50%, 80% or 90%, however, failed to significantly improve the performance of parsimony analysis (see Additional File 2: Table S2).

To report the details of our simulation we have organized the results to feature the input tree topology and tree-building algorithm. The four major variables tested in this simulation: tree topology, placement of reference taxon, tree building algorithm, and substitution rate, are discussed separately as much as possible given their inherent interrelationships.

### Balanced topology (Figure 3)

**Figure 3:**
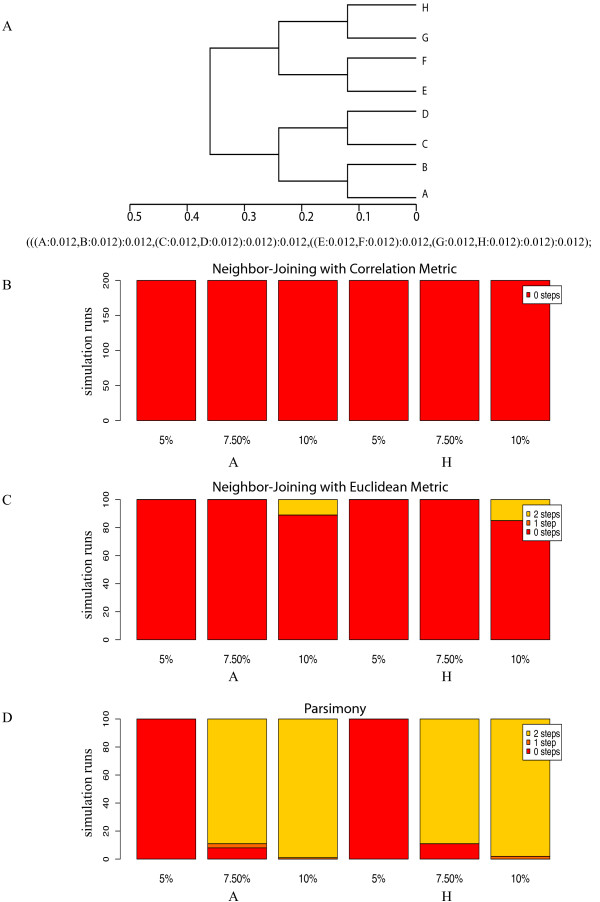
**Balanced Topology Results**. Figure 3A is a cladogram of an eight taxa balanced tree topology. In our simulation a sequence alignment was created using this input tree as a guide, where the branch lengths govern the number and pattern of substitutions. A number randomly drawn from a Gaussian distribution, empirically determined from published yeast genomic data, was used to multiply these branch lengths to produce a set of genes evolved for a range of substitution rates. The average of each range of substitution rates is indicated under each column. Figure 3B shows the symmetric distances for 200 simulation runs of the correlation-based NJ analysis evolved at the average sequence divergence indicated. Columns 1-3 use taxon A as the reference, Columns 4-6 use taxon H. Zero steps indicate perfect agreement between the input topology and the trees output by the simulation (red portions). One step away indicates a single collapsed branch (orange portions). A mispairing of two taxa is given a score of two (yellow portions). Scores in subsequent figures are cumulative and color-coded as indicated in the chart legends. Figure 3C shows the stacked histogram for the symmetric distances from the input topology for 100 replicate NJ trees calculated with the Euclidean distance metric. The order for reference taxa is the same as above. The stacked histogram in Figure 3D is of the symmetric distances from the input tree for 100 replicate 50% Majority-Rule Parsimony (PMR) trees. An example of this topology, two steps away from the reference, is given in Figure 4B.

Figure 3A shows an eight taxa tree in a balanced topology. The balanced topology is the simplest in terms of the relationships among the taxa because no matter which taxon is chosen as the reference, it will have a close sister taxon, a pair of taxa at a middle distance and two, most distant, taxon pairs. The three distances, sister, middle and distant, will not change as the reference taxon changes. Therefore, we expected to find no effect of choosing different reference taxa and, as can be seen in Figure 3B-D, the symmetric distances comparing input and output trees were identical when taxon A or taxon H was chosen as the reference taxon. This simple topology is recovered with moderate success with both distance-based NJ and parsimony tree construction but there are differences in the efficacy of the different tree construction algorithms and simulated substitution rates.

### NJ with the Pearson and Euclidean distance metric

When NJ was used with the Pearson correlation-based distance metric to make phylogenetic trees, comparison of input and output tree topologies by either symmetric distance or distance D1 showed no difference between the two topologies, regardless of nucleotide substitution rate (Figure 3B). When NJ was used with the Euclidean distance metric, there were discrepancies between the input and output topologies, but only at the highest rate of nucleotide substitution (Figure 3C).

### Parsimony Analysis

**Figure 4:**
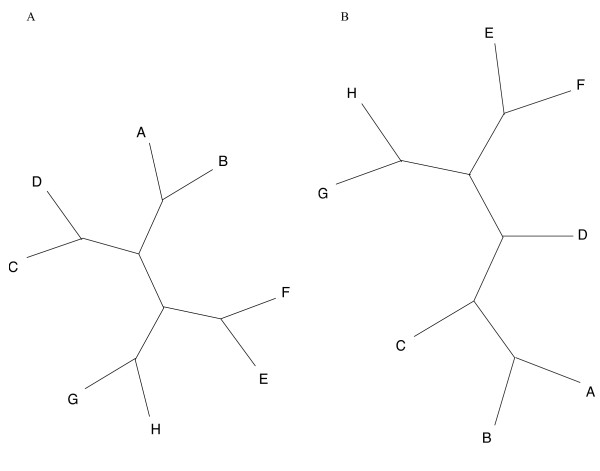
**The expected and recovered balanced topologies**. Figure 4A illustrates the eight taxa balanced topology as an unrooted cladogram. Figure 4B is the Euclidean NJ tree, when taxon A is the reference, as an unrooted cladogram and is two steps away according to the symmetric distance from the desired topology in Figure 4A. It was evolved at 7.5% average nucleotide substitutions.

With parsimony analysis (Figure 3D), substitution rate had a more dramatic effect. At 5% nucleotide substitution, there was no difference between the input and output topologies in all simulations, but, at substitution rates of 7.5% and 10% the input tree was recovered in no more than 8 of 100 simulations. At 7.5% and 10% sequence divergence, the pair of sister taxa closest to the reference taxon did not group together in more than 95% of simulations. Instead, these sister taxa collapsed to the base of the group as a polytomy or they resolved into separate lineages more than 95% of the time, as shown in the example in Figure 4A and 4B.

### Pectinate topology (Figure 5)

**Figure 5:**
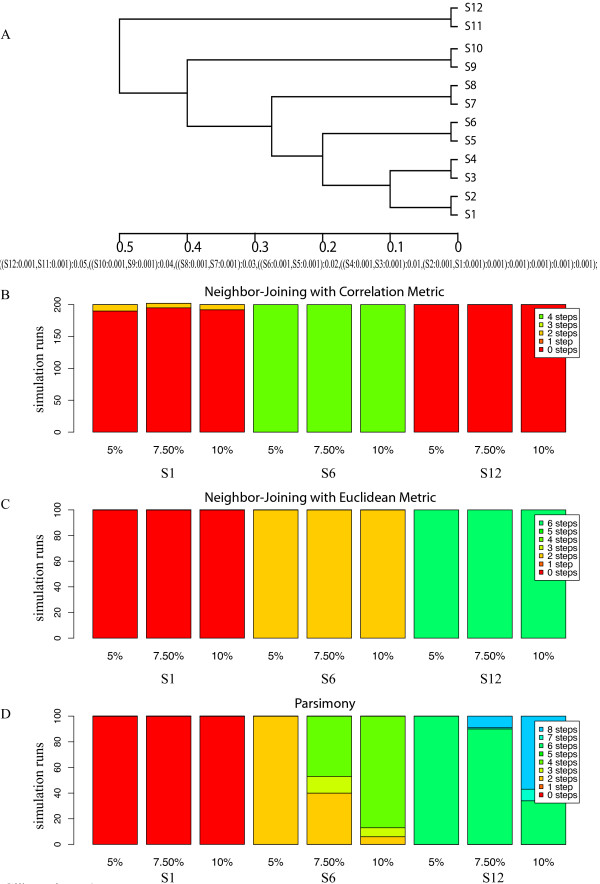
**Pectinate Topology Results**. Figure 5A shows the twelve taxa pectinate topology input into the simulation pipeline. Figure 5B shows the stacked histograms for the symmetric distances for 200 replicate correlation-based NJ. Columns 1-3 show S1 as the reference taxa, 4-6 show S6, and 7-9 represent taxon S12. Figure 5C give the stacked histogram for the symmetric distance for the Euclidean distance based NJ trees, 100 replicates. The order for reference taxa is the same as above. Figure 5D gives the corresponding stacked histogram for 100 replicate PMR trees. Two examples of this topology, six and eight steps away from the reference, are given in Figure 6B and 6C.

This 12-taxon tree (Figure 5A) shows a modified pectinate form in which at most four taxa have the same relationships to the other members of the tree, i.e., S1 to S4. Each of these four taxa has a close sister taxon and then shows increasingly longer distances to four other taxon pairs. This situation is in marked contrast to the balanced tree in Figure 3A, where all members had the same relationships to the other members. To assess the effect of the position of the reference taxon, we designated three different taxa as the reference, S1, S6, and S12 (Figures 5B, C, and 5D).

### NJ with the Pearson and Euclidean distance metric

The simulations show that the pectinate topology is not recovered as frequently as the balanced topology. NJ using the Pearson correlation-based distance recovered the input tree most frequently, with almost 2/3 of the trees 0 steps away. Choice of the reference taxon had a strong effect with the pectinate tree. For example, with NJ using Pearson's correlation distances, the input tree was recovered at least 95% of the time when the most basal (S12) or the most derived taxon (S1) were used as reference, but never when an interior taxon (S6) was designated as reference (Figure 5B).

With NJ using Euclidean distances, the input topology was recovered in 100% of simulations when the most derived taxon was the reference taxon, but almost never when either an internal (S6) or basal (S12) taxon was designated as reference (Figure 5C). With NJ using Euclidean distances and with parsimony, input trees were recovered 100% of the time when the most derived taxa were the reference, but never when either internal or basal taxa were designated the reference taxon. In fact, use of the basal taxa as reference made the output trees at least 6 steps different from the input trees with both NJ using Euclidean distance or parsimony.

### Parsimony Analysis

**Figure 6:**
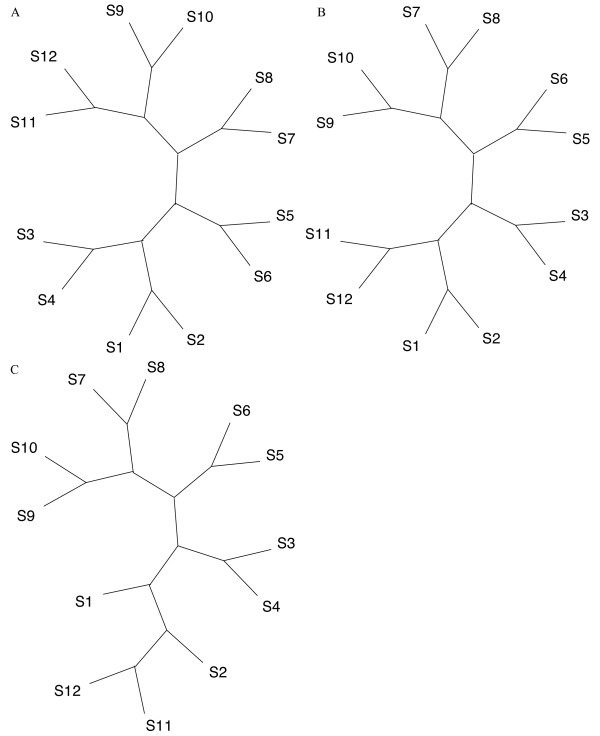
**The expected and recovered pectinate topologies**. Figure 6A shows the twelve taxon pectinate topology drawn as an unrooted cladogram. 6B show an example where S12 acts as the reference for the NJ Euclidean method, it is 6 steps away according to the symmetric distance from the reference tree in A. C shows another example tree where S12 is the reference but for the parsimony algorithm, and is eight steps away. Both 6B and 6C were evolved at 7.5% average nucleotide substitutions.

With parsimony analysis, the input topology was recovered in all simulations when the most derived taxon was the reference, but never when an internal or basal taxon was so designated (Figure 5D). The parsimony analysis (Figure 5D) behaved very similarly to the Euclidean-based NJ analyses (Figure 5C). However, parsimony analysis was marginally worse than NJ because taxa S1 and S2 occasionally collapsed into a polytomy with the S3-S4 pair, Which increased the number of steps between input and output trees (SymD), as did higher substitution rates. Example trees are given in Figure 6.

### *Neurospora *topology (Figure 7)

**Figure 7:**
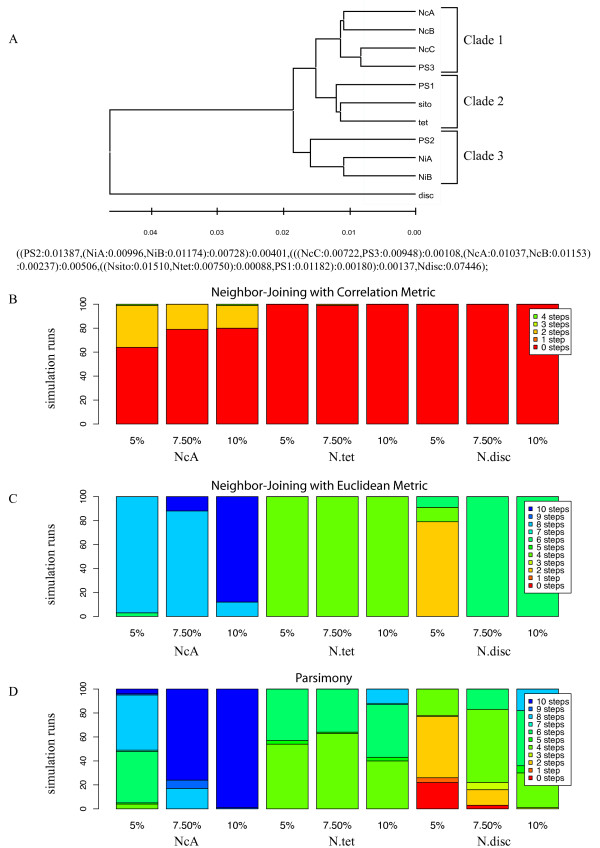
**Neurospora Topology Results**. Figure 7A gives the topology of the natural phylogeny for the eight conidiating species of *Neurospora*, modified from Dettman 2003a. Figure 7B gives the stacked histogram shows the proportion of NJ trees in 200 replicates that are 0 or more steps away from the input topology. The first three columns show the symmetric distance using *N. crassa *clade A (NcA) as the reference taxa for the average sequence divergence indicated. Columns 4-6 show the results using *N. tetrasperma *(tet) as the reference taxa. Columns 7-9 use *N. discreta *(disc) as the reference. Figure 7C gives the stacked histogram of the symmetric distance for 100 NJ trees built with the Euclidean distance metric. No trees were less than 2 or more steps away. Figure 7D gives the stacked histogram showing the proportion of PMR trees in 100 replicates that are 0 or more steps away from the input topology. Three examples of this topology, between four to eight steps away from the reference, are given in Figure 8B, 8C, and 8D.

The *Neurospora *phylogeny is based on observed distances among species in this genus and is used here as an example of a natural topology to begin the simulation process [24]. This 11 taxon phylogeny combines elements of the balanced and pectinate topologies (Figure 7A in that it contains a mixture of close and distantly related taxa, some of which are connected with small internal branches. These features present a challenge for array CGH tree-building algorithms.

As with the other topologies, we evaluated the effects of choosing different reference taxa, *N. discreta, N. crassa *and *N. tetrasperma*, in addition to the effects of different methods of phylogenetic analysis and different rates of nucleotide substitution. In summary, the phylogenetic method had the strongest effect on recovery of the input topology. NJ with Pearson's correlation (Figure 7B) performed far better than either the NJ with Euclidean distance or parsimony methods. Neighbor-Joining with Euclidean distances (Figure 7C) never recovered the input phylogeny and with parsimony (Figure 7D), the input tree was almost never recovered.

### NJ with the Pearson and Euclidean distance metric

With NJ using Pearson's correlation (Figure 7B), designating the basal taxon (*N. discreta*) as reference resulted in recovery of the input tree 100% of the time. The same result was seen when *N. tetrasperma *was designated reference, but only 64% of simulations did when *N. crassa *A was designated reference. Instead, output trees were occasionally two to four steps different from the input tree, resulting from a failure to resolve short internal branches separating branches among taxa within clade 2 and by placing the long branch of *N. discreta *incorrectly. When *N. tetrasperma *or *N. discreta *was the reference, these short connective branches were resolved almost 100% of the time. When the nucleotide substitution rate was increased from 5% to either 7.5% or 10%, a greater percentage of simulations recovered the input topology, suggesting that more polymorphisms help differentiate those short branches for this distance metric.

**Figure 8:**
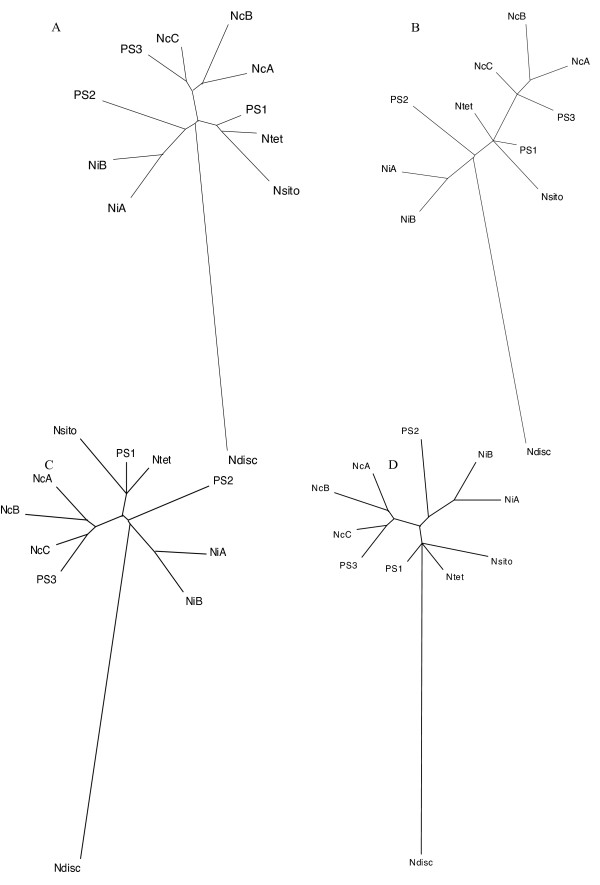
**The expected and recovered *Neurospora *topologies**. Figure 8A is the *Neurospora *topology drawn as an unrooted phylogram. Figure 8B shows an example were *N. crassa A *is the reference for the Euclidean NJ that is 8 steps away from A according to the symmetric distance. Figure 8C shows an example with *N. tetrasperma *as the reference for the Euclidean NJ that is 4 steps away from the reference according to the symmetric distance. Figure 8D shows an example with *N. discreta *as the reference that is 6 steps away according to the symmetric distance by the Euclidean NJ method. B, C, and D were evolved at 7.5% average nucleotide substitutions.

With NJ using Euclidean distance, the input topology was never recovered and the majority of output trees were 4 or more steps different from the input topology (Figure 7C). In these trees, many sister taxa relationships devolved into polytomies when small internal supporting branches were left unresolved. When *N. discreta *was designated as the reference taxon, a higher proportion of CGH trees were more similar to the input tree - especially when the nucleotide substitution rate was lowest. *N. crassa *A performed most poorly as the reference with most output trees at least 6 and as many as 10 steps distant from the input tree (Figure 8A and 8B). When *N. tetrasperma *was the reference, the short internal branches remained unresolved, and when either *N. crassa *A or *N. tetrasperma *were the reference taxon, there was a tendency to erroneously group *N. discreta *with clade 3 (Figure 8C). At low mutation rates, *N. discreta *and *N. crassa *A performed better as the reference, although the discrepancies between the input and output trees worsened as the substitution rates increased. (Figure 8D). Close examination of these trees suggests that a saturation of polymorphisms made shorter branches difficult to resolve.

### Parsimony Analysis

With parsimony, again the input tree was recovered only when the most basal taxon, *N. discreta*, was reference and then only 22% of the time at 5% nucleotide substitution and only 3% of the time at 7.5% nucleotide substitution. When *N. crassa *A or *N. tetrasperma *were designated as reference taxa, parsimony analysis was unable to correctly place the most divergent taxon, *N. discreta*. For these trees, *N. discreta *was incorrectly placed in a polytomy within clade 3 instead of outside of it. With increasing sequence differences, *N. discreta *was placed within clade 2 or between clades 1 and 2 (Additional File 3: Figure S1).

### Probe Length

Increasing the length of the probe sequence in the microarray from 70 nt to 500 nt might be expected to improve the recovery of input topologies by providing more polymorphisms for phylogenetic analysis. To test this hypothesis, 25 replicate runs at 10% average sequence divergence were completed using 500 bases as the length of the probe for the Neurospora topology. There was no significant improvement in recovery of the input topology regardless of which taxon was designated as the reference taxon with the longer alignments (Additional File 2: Table S2).

### Cophenetic Correlations

**Figure 9:**
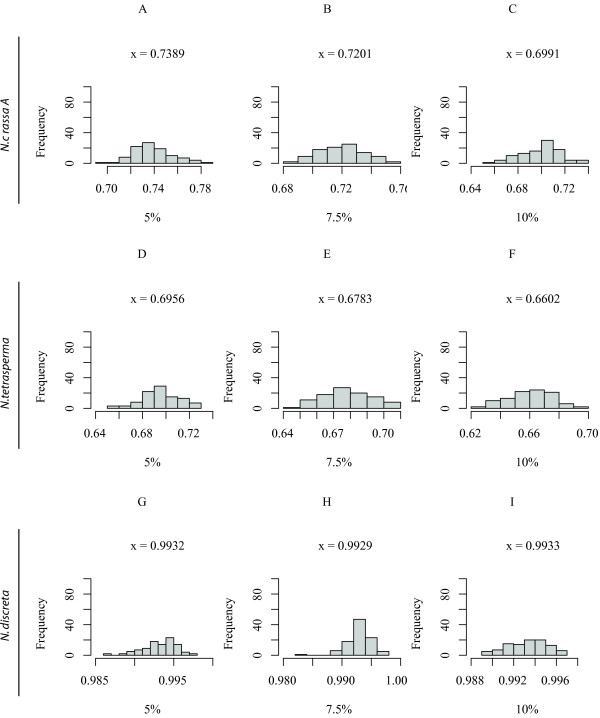
**The cophenetic correlation of the correlation-based NJ for the *Neurospora *topology**. The distribution of the cophenetic correlations for Tree 3 using the correlation-based NJ trees. Figures 9A, 9B, and 9C give the distribution of correlations using *N. crassa A *(NcA) as the reference for the average sequence divergence shown. Figures 9D, 9E, and 9F use *N. tetrasperma *(tet) as the reference taxa. Figures 9G, 9H, and 9I represent reference taxon *N. discreta *(disc). The average correlation for each set replicates is shown with each distribution.

**Figure 10:**
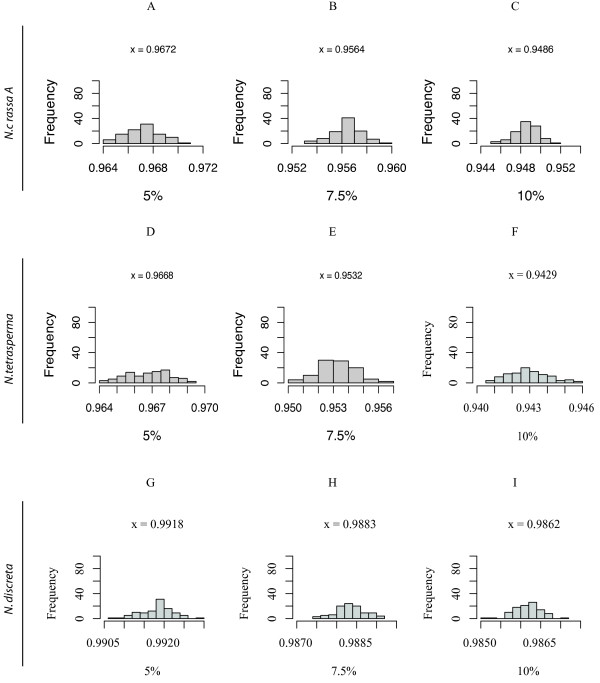
**The cophenetic correlation of the Euclidean-based NJ for the *Neurospora *topology**. The distribution of the cophenetic correlations for Tree 3 for 100 replicate Euclidean distance based NJ Trees. Histograms 10A, 10B, and 10C are *N. crassa A *(NcA) reference based. Histograms 10D, 10E, 10F are *N. tetrasperma *(tet) reference based. Histograms 10G, 10H, and 10I represent reference taxon *N. discreta *(disc). Note that the correlations are high, because of the correspondence in branch lengths even though the topologies aren't similar.

To evaluate differences in the branch lengths, irrespective of topology, of the input and output trees obtained by NJ using Pearson's correlation or Euclidean distances, we determined the cophenetic correlation coefficient (CCC) for the *Neurospora *distance trees (Figures 9 and 10). When assayed by topology, NJ with Pearson's correlation was more successful than the Euclidean in finding the topologically correct tree. However, when assayed by the CCC, the Euclidean method was better at estimating the branch lengths. This result was true not only for the *Neurospora *tree, but also for the pectinate tree. For the balanced tree, the CCC was slightly better for the NJ using Pearson's correlation than with Euclidean distance. The CCC also varied moderately with the choice of reference taxon and slightly with the percent substitution rate. Distributions for the other topologies are included in the Additional Files 4, 5, 6, and 7: Figures S2, S3, S4, and S5.

## Discussion

Array Comparative Genomic Hybridization (array CGH) is a technique in which the genomic DNAs of a reference individual and a close relative are hybridized to a microarray made of probes designed to match the genome of the reference individual. To assess the appropriateness of using microarray data for phylogenetic analysis, we asked if having a single reference species, to which all other taxa must be compared, limits the ability to accurately recover evolutionary relationships. In our simulations, to make phylogenies from CGH data, we used both distance and parsimony phylogenetic methods. We assessed the effects of tree topology, the position of the reference taxon in the topology, the rate of nucleotide substitution, and the length of microarray probes. We assessed the difference between the input and output phylogenies using two tree-to-tree distance metrics and also assessed the difference in rates of evolution for two topologies as measured from branch length differences.

Our results show that under specific conditions, using CGH to quantify inter-genomic sequence variation can yield data that support the input topology. However, it is just as common to get a tree with little resemblance to the true tree. The user would be unaware of this, as studies often have no prior knowledge of two key parameters, the topology and the position of the reference taxon. Unfortunately, these are the goals that phylogenetic inference is designed to determine. CGH, however, may have a role to play in assessing sequence divergence, by identifying genes that are evolving rapidly, but that are still present across taxa, that would be good candidates for multi-locus sequence analysis.

## Summary of Simulation Results

Tree topology and the position of the reference taxon had the greatest effects on successful recovery of the input tree by CGH. The balanced topology was recovered more successfully than the pectinate or *Neurospora *topology, probably because all taxa occupy the same position relative to their neighbors in the balanced topology. For the pectinate topology, CGH was most successful with reference taxa in the most derived position. This result is not unexpected, as the single reference design of most CGH arrays constricts the dataset in such a way that discards information that would resolve irregular distances. In effect it is similar to constricting multi-dimensional scaling data to a single plane- that in this case is dictated by the choice of reference taxon. For the *Neurospora *topology, CGH success was poorest with the reference in a derived position, but for this topology the analytical method had a stronger influence than the position of the reference taxon.

In general, the NJ distance method was more successful at recovering the input topology than was parsimony, and distance data matrices made using Pearson's correlation coefficient performed better than those made by Euclidean distance. For the correlation, in the tree-building process it was found to be more expedient to exclude the reference as the correlation method was not able to cope with the reference taxon's lack of variability in the evolved data. However, branch lengths were better recovered with NJ using a Euclidean distance matrix than with the Pearson's coefficient. While the Pearson's correlation is more robust to missing data and is less sensitive to small variations than Euclidean distance, the Euclidean potentially preserves more information about the genetic distance [59].

For parsimony analysis all "species" hybridization values were binned using a single cutoff, a simple approach we found adequate for distribution of sequence polymorphisms simulated here. Modifying the threshold for assigning shared character states (1,1) from the 75^th ^percentile to the 80^th ^percentile or higher occasionally improved the results, but only for some locations of the reference taxon and topologies.

Varying the rate of nucleotide substitution had little effect on recovery of the input topology by CGH, although at high substitution rates parsimony analysis performed better when the threshold was high for scoring a hybridization spot as absent. Increasing the length of the probe from 70 bases to 500 bases had no substantial effect on recovery of the input topology.

Of particular importance to a correct CGH phylogeny is the choice of taxa included in the analysis. Taxa with reasonable distance to each other, avoiding mixtures of close and more distantly related taxa, are more easily captured. It is also best to have a reference taxon in the most basal position of the tree, or second-best the most derived position. *A priori*, these two parameters are never known except in test cases, making CGH problematic for practical use in phylogenetics.

### Comparison to other studies using CGH to infer phylogenetic relationships

Initial studies of CGH for phylogeny include research with *Salmonella typhimurium *and *Bordetella pertussis *microarrays. For the *Bordetella *study a parsimony CGH tree has at least one major difference when compared to the available MLEE tree [39], the latter being an unreliable method as it only determines phenotypes and is not very reproducible. NJ and Parsimony CGH trees for *Salmonella *are also at least one step away from several sequence trees used for comparison [60].

Other researchers utilizing *Bacillus, Streptococcus*, and *Ralstonia *microarrays report congruence between CGH-derived topologies constructed using hierarchical clustering, NJ, or Parsimony methods of bacterial clusters or species groups and DNA sequence trees based on several loci or MLEE [41,61,62]. All of these studies highlight the similarities of their trees to the previously published work but say little about the differences of their CGH-based trees to those derived via more traditional methods though the *Streptococcus *study found genetic relatedness only within clonal complexes, not between them. Though one or two branch rearrangements may not be substantial difference to the researcher, it demonstrates the limitations of this phylogenetic technique by failing to resolve a perfect tree.

It is possible that a CGH phylogeny from a multi-species array would better reflect evolutionary distances among taxa. While answering this question is outside the scope of this study at least one other, Wan et al [44], has addressed it. Unlike our study, which focused on a single species array, Wan et. al analyze the effects of the composition of a multi-species array and propose a bias-correction algorithm for uneven species content. Using both experimental and simulated data for a mixed *Enteroccocus *species array they find a CGH tree one step away from a 5 gene MLST tree of the same species. In their *in silico *study they see longer branch lengths from reference to other taxa in reconstructed trees, as we do, but stress that the clustering of groups should be unaffected in most cases.

In a more recent study aCGH data from bovine and human data as well as simulated data were examined by the developers of a wavelet based de-noising algorhythm aimed at quantifying structural variation at the genome level [38]. Though the authors restricted their study of evolutionary relatedness to clusters of intra-species data they expressed an interest in future phylogenetic analysis utilizing their algorithm as a starting point. The large scale structural variations simulated by the authors were quite different from the closely related relationships we chose to model in this work. This different approach to the same question did not address the factors we found most pertinent - the topology of underlying tree and choice of reference taxon.

## Conclusions

Our results show that CGH cannot be counted on to reveal genomic sequence divergence reliably enough to recover a known phylogeny. Consequently, relying on this technique to recover an unknown phylogeny is problematic, particularly when there is no traditional phylogeny to compare to or when an existing phylogeny is based only a few genes or obsolete techniques like MLEE.

The results from our *in silico *study presented here are further substantiated by a thorough analysis of experimental data in our accompanying manuscript and by the various pro-CGH phylogeny papers enumerated above which detail minor, but significant, differences between their trees and previously published works.

Given the drawbacks of CGH for phylogeny, resulting in high uncertainty in the correct topology, it would be perhaps be more prudent to utilize the cross-taxa CGH data to identify a set of genes suitable for MLST, those still present but with a high to moderate amount of variation, then pursue a traditional phylogenetic analysis.

## Authors' contributions

LBG participated in the simulation design and coordination and drafted the manuscript. LC, TK, and JWT participated in the design of the study. LC and LBG scripted code for the completion of the simulation. LBG completed the statistical analysis. All authors read and approved the final manuscript.

## Supplementary Material

Additional file 1 **Additional Table S1**. Tree to Tree distances for all Neighbor-Joining Results. Click here for file

Additional file 2 **Additional Table S2**. Tree to Tree distances for all Parsimony Results. Click here for file

Additional file 3 **Additional Figure S1**. Example topologies of Neurospora Parsimony simulation results in pdf format. Click here for file

Additional file 4 **Additional Figure S2 Correlation-Based CCC distributions**. Balanced topology Cophenetic Correlations Coefficient Distributions for the correlation-based Neighbor-Joining Analysis in pdf format. Click here for file

Additional file 5 **Additional Figure S3 Euclidean-Based CCC distributions**. Balanced topology Cophenetic Correlations Coefficient Distributions for the Euclidean-based Neighbor-Joining Analysis in pdf format. Click here for file

Additional file 6 **Additional Figure S4 Correlation-Based CCC distributions**. Pectinate topology Cophenetic Correlations Coefficient Distributions for the correlation-based Neighbor-Joining Analysis in pdf format. Click here for file

Additional file 7 **Additional Figure S5 Euclidean-Based CCC distributions**. Pectinate topology Cophenetic Correlations Coefficient Distributions for the Euclidean-based Neighbor-Joining Analysis in pdf format. Click here for file
