# Supplementary figures and images for: Array Comparative Genomic Hybridizations: Assessing the ability to recapture evolutionary relationships using an in silico approach
Source: BMC Genomics. 2011 Sep 21;12:456. doi: 10.1186/1471-2164-12-456 (PMC3196971; doi:10.1186/1471-2164-12-456)

A

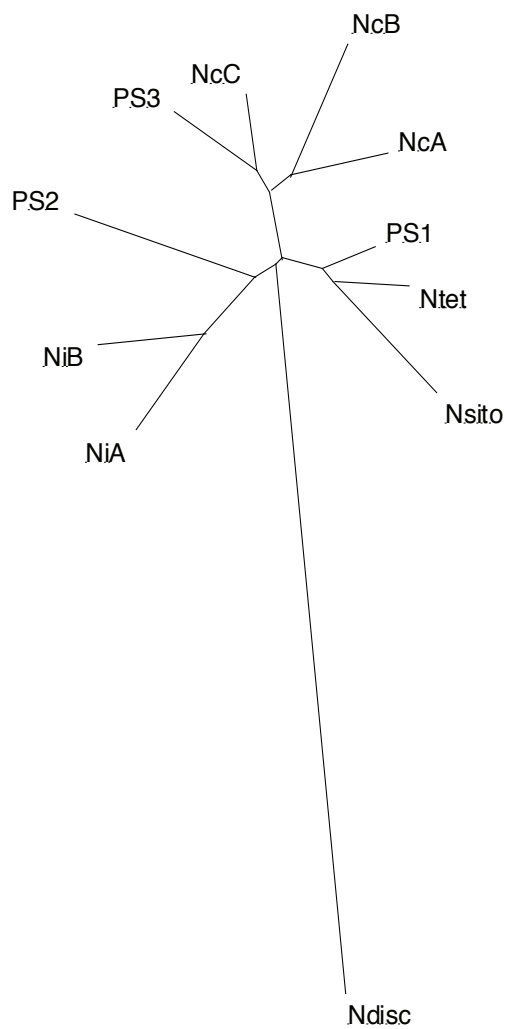

B

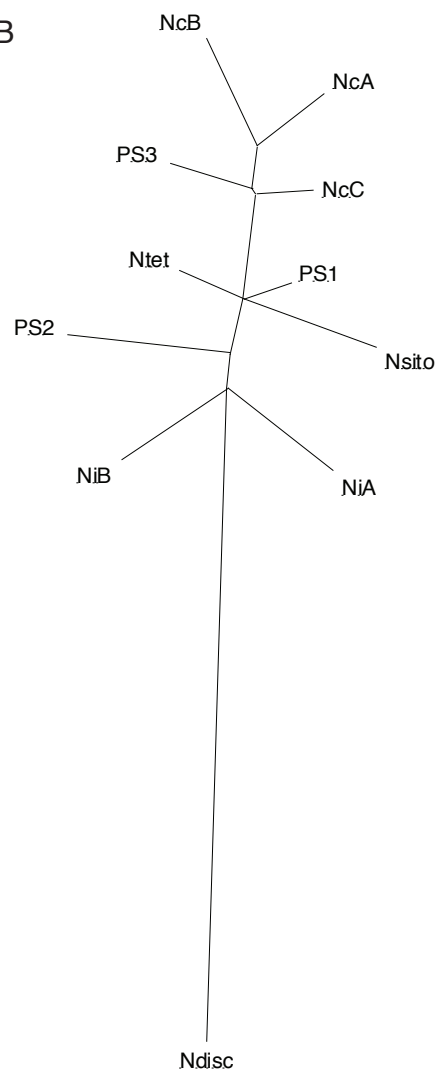

C

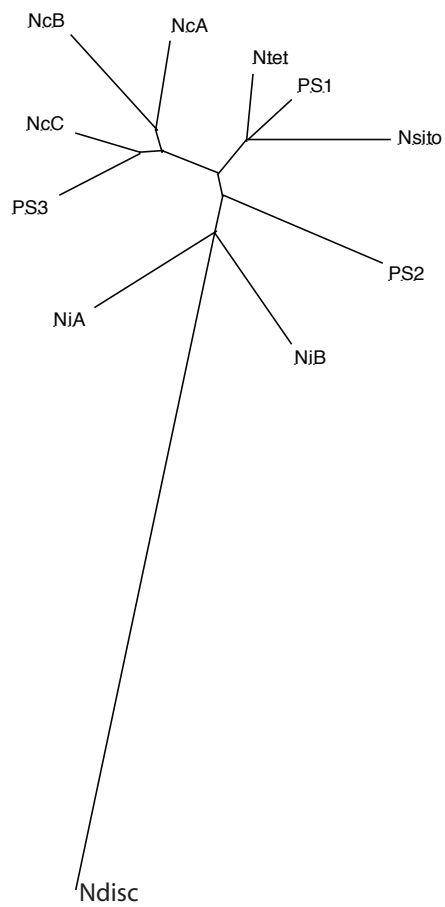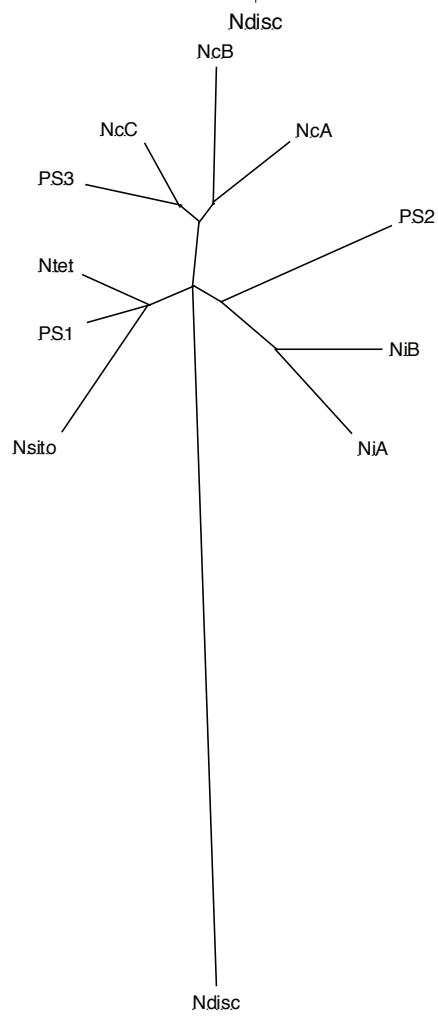

Supplement: Additional file 3 — Additional Figure S1. Example topologies of Neurospora Parsimony simulation results in pdf format. [file 1471-2164-12-456-S3.PDF]

T3\_correlation vs og input tree  
ABC taxa A  
DEF taxa H

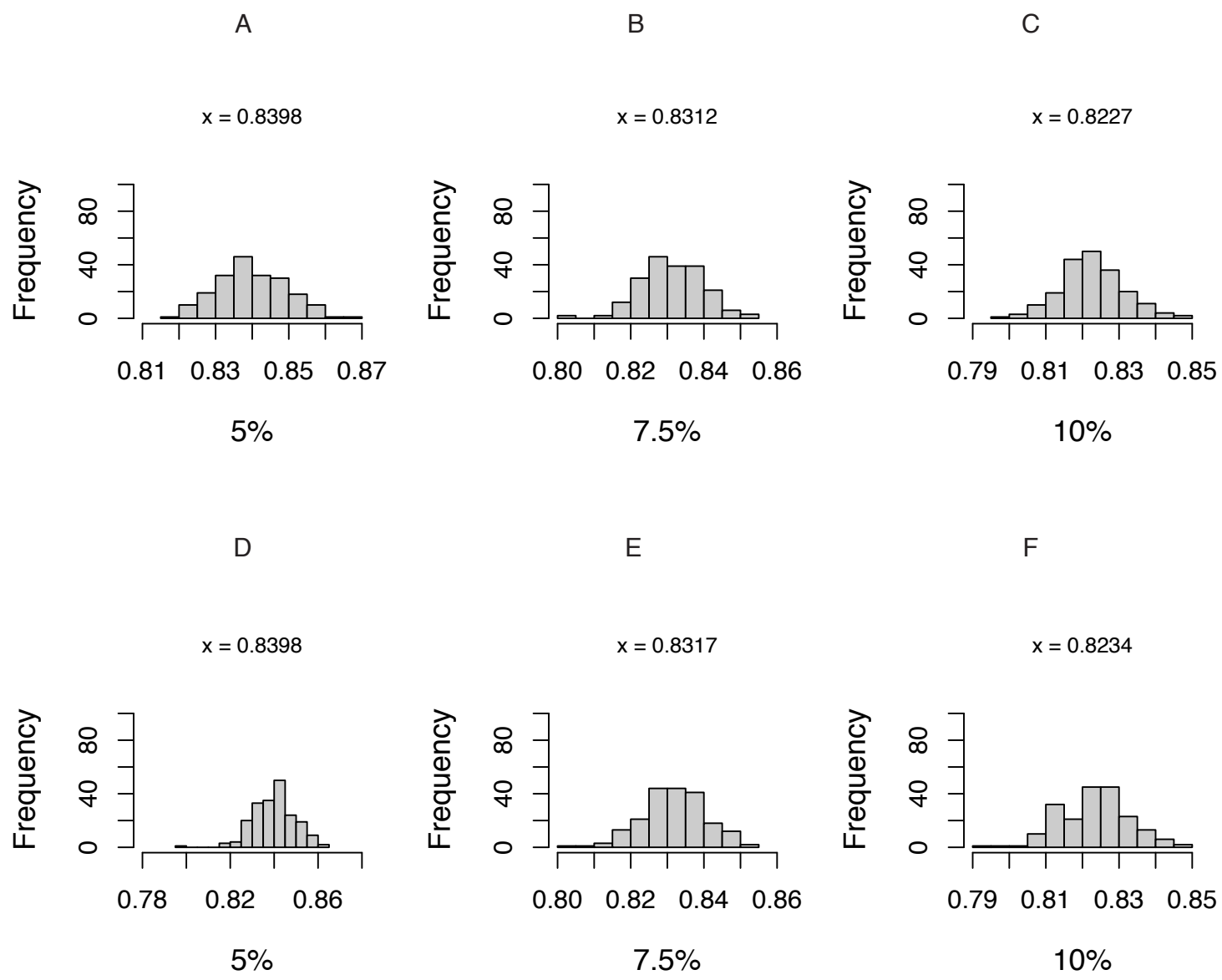

Supplement: Additional file 4 — Additional Figure S2 Correlation-Based CCC distributions. Balanced topology Cophenetic Correlations Coefficient Distributions for the correlation-based Neighbor-Joining Analysis in pdf format. [file 1471-2164-12-456-S4.PDF]

T3\_euclidean vs og input tree  
ABC taxa A  
DEF taxa H

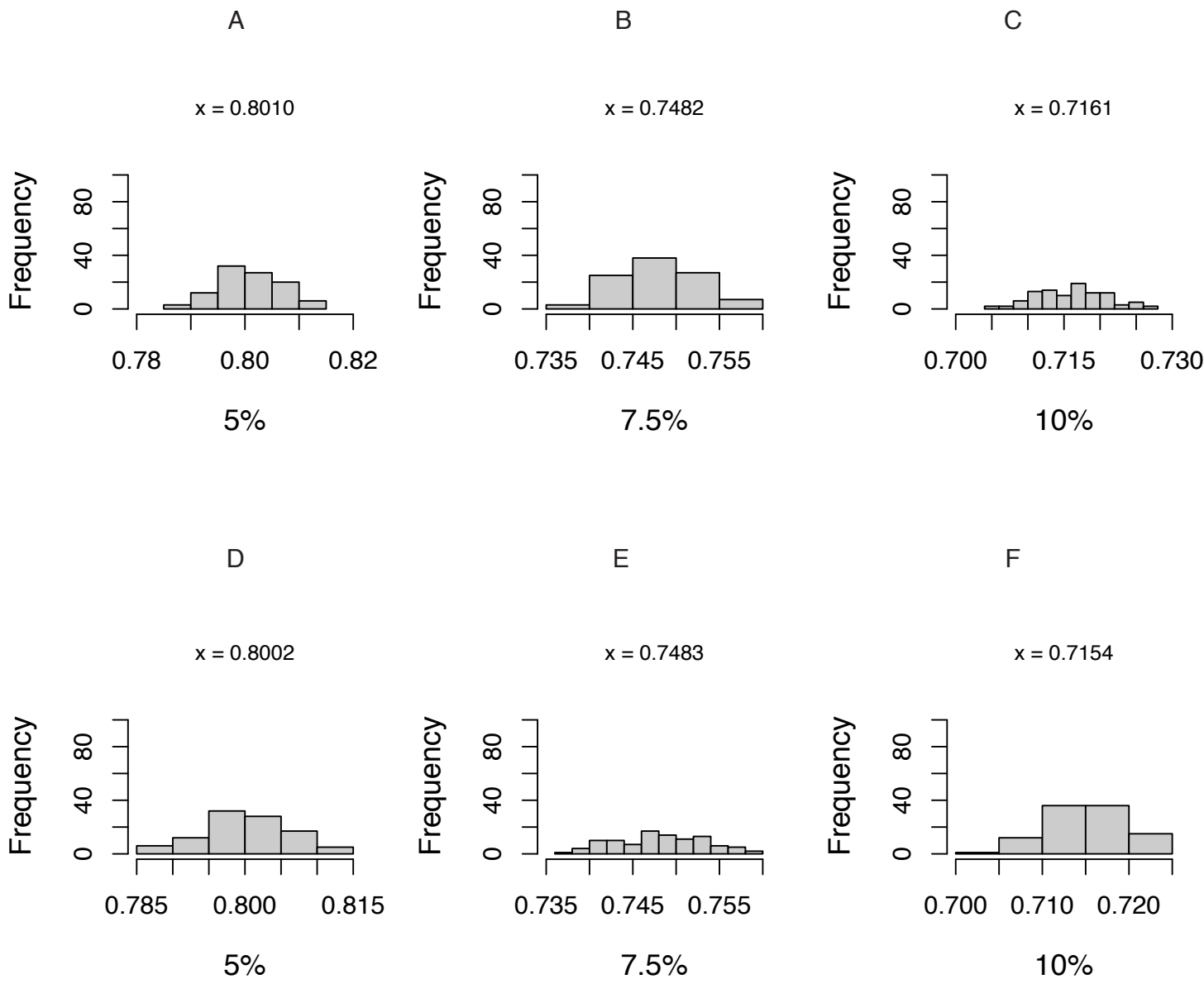

Supplement: Additional file 5 — Additional Figure S3 Euclidean-Based CCC distributions. Balanced topology Cophenetic Correlations Coefficient Distributions for the Euclidean-based Neighbor-Joining Analysis in pdf format. [file 1471-2164-12-456-S5.PDF]

T2 Correlation vs o.g, tree  
ABC S1  
DEF S6  
GHI S12

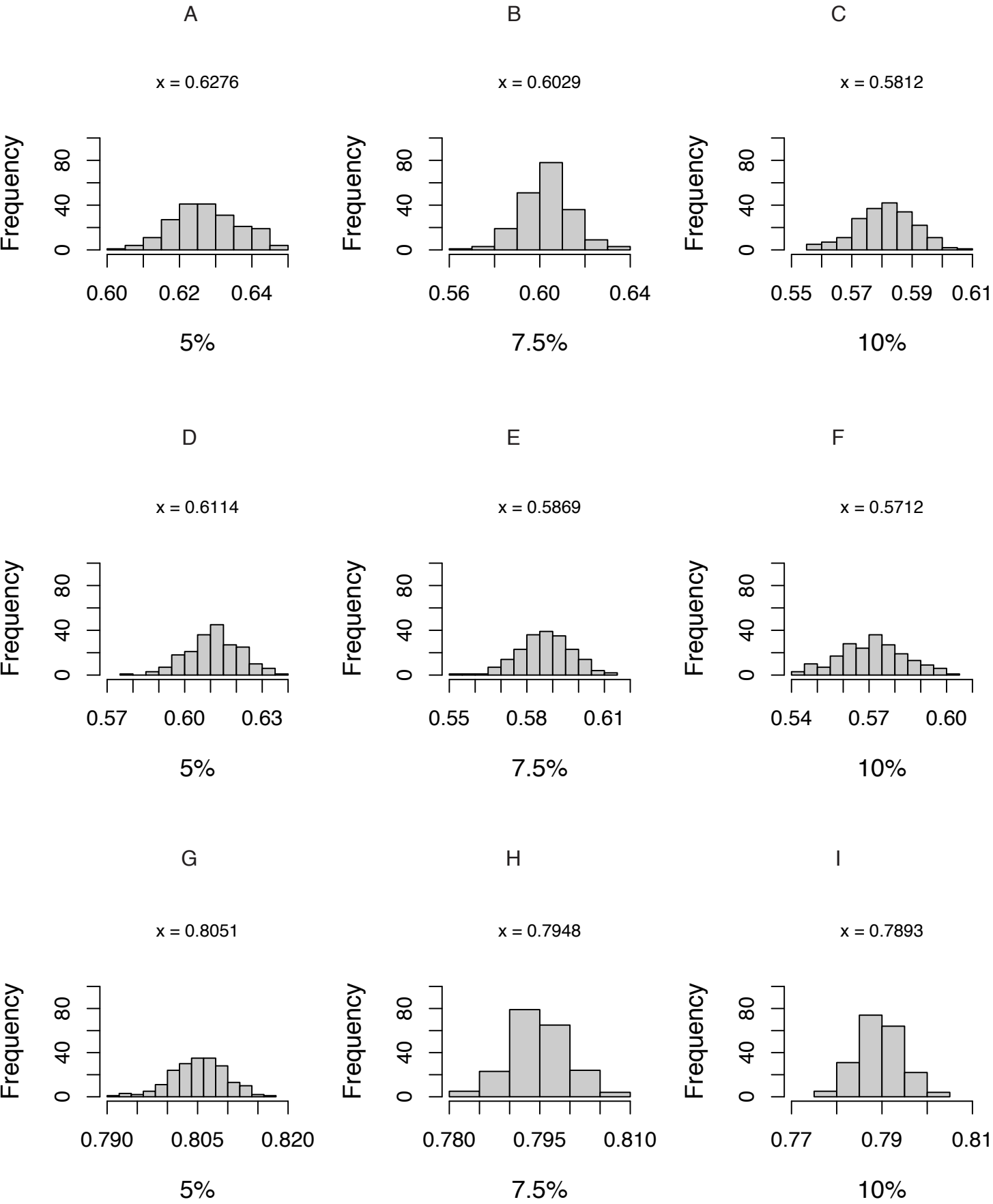

Supplement: Additional file 6 — Additional Figure S4 Correlation-Based CCC distributions. Pectinate topology Cophenetic Correlations Coefficient Distributions for the correlation-based Neighbor-Joining Analysis in pdf format. [file 1471-2164-12-456-S6.PDF]

T2 Euclidean vs o.g. tree

ABC S1

DEF S6

GHI S12

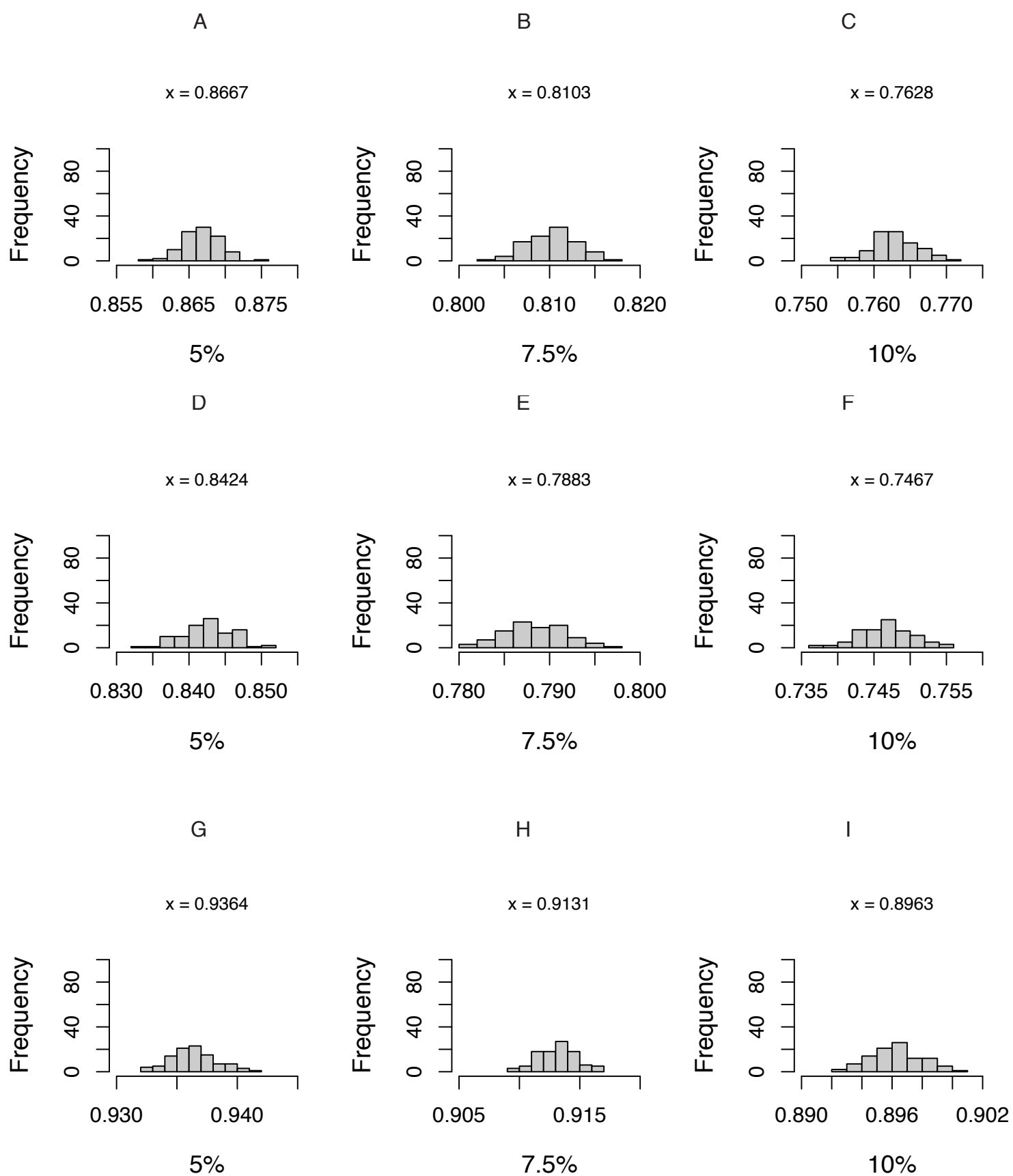

Supplement: Additional file 7 — Additional Figure S5 Euclidean-Based CCC distributions. Pectinate topology Cophenetic Correlations Coefficient Distributions for the Euclidean-based Neighbor-Joining Analysis in pdf format. [file 1471-2164-12-456-S7.PDF]
